# Perceived support and psychological resilience as collaborative mediators between external support and quality of life in elderly breast cancer patients

**DOI:** 10.1038/s41598-025-23246-x

**Published:** 2025-11-11

**Authors:** Jihong Wei, Zhuoxin Yang, Xiaohong Chen, Lingyun Tan, Haiyan Hu, Xiaohong Zhang, Zhuowei Tang, Yuwei Yang

**Affiliations:** 1 School of Medicine, Mianyang Central Hospital, University of Electronic Science and Technology of China, Mianyang, China; 2 Nursing Department, Jiange People’s Hospital, Guangyuan, China; 3 Nursing Department, Shanxi Traditional Chinese Medical Hospital, Taiyuan, China

**Keywords:** Elderly, Breast cancer, Social influence, Perceived social support, Resilience, Quality of life, Human behaviour, Health services, Quality of life

## Abstract

Both patients’ psychological resilience and perceived social support significantly moderate the impact of external social support on quality of life (QoL), yet their interactive contribution remain largely unclear. This study focuses on elderly breast cancer patients, and aims to investigate how these factors individually and interactively mediate the effect of social support on QoL. A total of 143 elderly breast cancer patients were recruited in this cross-sectional study, to survey the Social Impact Scale (SIS), Perceived Social Support Scale (PSSS), Breast Cancer Resilience Scale (BCRS), and Short Form-36 Health Survey (SF-36). The independent associations between SF-36 scores with other scale dimensions were investigated in particular, both unadjusted and adjusted for adjusting for demographic characteristics. Furthermore, the individual and interactive effects of PSSS and BCRS in the relationship between SIS and SF-36 were systematically evaluated. SF-36 overall and dimensional scores exhibited generally negative correlations with SIS scores (most r≤-0.3, P < 0.001), while generally positive correlations with PSSS and BCRS scores (most r ≥ 0.3, P < 0.001). Before adjusting for demographic characteristics, SIS3-social isolation dimensional score demonstrated negatively independent association with SF-36 overall and dimensional scores (β=-0.82~-13.50, all P < 0.05), whereas BCRS1-personal protection dimensional score demonstrated positively independent associations with SF-36 overall score and its “general health”, “vitality”, and “mental health” dimensional scores (β = 0.64 ~ 5.74, all P < 0.05), and PSSS1-family support score demonstrated positively independent associations with SF-36 “social function” and “mental health” dimensional scores (β = 0.93 and 1.27, both P < 0.05). The independent association analysis incorporating demographic characteristics revealed prediction roles of BCRS1-personal protection, SIS3-social isolation, patient age, and disease duration on the SF-36 overall score (all P < 0.05). Finally, our mediating effect model revealed that neither PSSS nor BCRS alone significantly mitigated the effect of SIS on SF-36 (both P > 0.05). Instead, PSSS mitigated this effect by enhancing BCRS, contributing 8.39% in total effect (P < 0.05). Our findings suggest that patients’ perception of external social support improves their QoL by enhancing their resilience. Notably, external social isolation press and patients’ personal protection psychology are closely associated with their overall QoL.

**Supplementary Information:**

The online version contains supplementary material available at 10.1038/s41598-025-23246-x.

## Introduction

 Breast cancer is one of the most common malignant tumors among women, with an incidence rate as high as 52.81/100,000 and a mortality rate of approximately 13.40/100,000^1^. It has become one of the malignant tumors that pose a serious threat to human life and health^2^. In recent years, with the global aging trend, the number of elderly breast cancer patients aged over 60 has shown a gradual upward trend^3^. Owing to the continuous progress in breast cancer diagnosis and treatment technology, the 5-year relative survival rate of female breast cancer patients has reached 73.0% to 80%^4^, which is significantly higher than that of other types of malignant tumors. Therefore, paying attention to the physical and mental health of breast cancer patients has become an important hotspot issue of health care.

For elderly breast cancer patients who are eligible for surgery, surgery intervention represents one of the preferred treatment options^5^. However, surgical resection not only brings significant physical pain to the patients but also may cause them to bear psychological pressure from multiple aspects such as family and society. It directly destroys their female image characteristics, challenges their identification with the traditional female body image, and even may have adverse effects on their mental health, thereby seriously interfering with their quality of life (QoL)^6^. Furthermore, for elderly breast cancer patients without surgical indications, the cancer diagnosis itself can substantially elevate their psychological burden, causing fear and uncertainty^7^. Even with breast preservation, anxiety and depression may arise from the impact of disease or treatment (e.g., radiotherapy, chemotherapy) on body image, worsening psychological burden, and affecting physical function and QoL^8^. Therefore, irrespective of whether surgical resection treatment is feasible, the QoL of elderly breast cancer patients is compromised by their inherent concerns and fears. Simultaneously, these emotional burdens are transferred to their families and society, thereby evolving into a multidimensional issue encompassing psychological, physiological, economic, and social aspects.

Previous studies have shown that psychological resilience can maintain the mental health of vulnerable people, mitigating their negative emotions such as anxiety and depression, and enhancing their overall QoL^9^. Current evidence suggests that psychological resilience plays a significant role in enhancing the QoL among breast cancer patients^10,11^. This resilience arises not only from intrinsic patient factors but is also shaped by family and social support. Numerous scholars, both domestically and internationally, have identified social support as a crucial protective factor during the development of psychological resilience^12^. Social support is considered a pivotal mediator for maintaining mental health and positively influences the psychological adjustment of cancer survivors^13–15^. Particularly for breast cancer patients, alterations in body image substantially augment their physiological and psychological burdens while reducing social adaptability^16,17^. However, the influence of social support on psychological resilience and their combined roles in enhancing the QoL among breast cancer patients remain underreported. Therefore, investigating the combined mediating effects of perceived social support and psychological resilience in the relationship between social influence and QoL in elderly breast cancer patients holds considerable significance for improving their overall well-being.

According to these situations, the present study designs a cross-sectional survey to investigate the perceptions of social support, social influence, psychological resilience, and QoL among elderly breast cancer patients and explores the correlations between these factors. This can provide a scientific basis for developing more effective perioperative interventions.

## Materials and methods

### Ethical review

This cross-sectional study was in accordance with the Declaration of Helsinki principles and STROBE guidelines for cross-sectional study, and approved by the Ethics Committee of Mianyang Central Hospital (S20240223-01). All participants signed the informed consent form on the title page of our designed *Wenjuanxing* questionnaire.

### Participants

The elderly breast cancer patients hospitalized in the Breast Disease Center between October 2023 and March 2024 were recruited for the investigation. The inclusion criteria were as follows: (1) Patients aged ≥ 60 years old; (2) Diagnosis confirmed by pathological examination and met the criteria of “Chinese Anti-Cancer Association Breast Cancer Diagnostic and Treatment Guidelines and Norms (2021 Edition)”^18^; (3) Patients with clear expression and comprehension abilities; (4) Patients fully understood their condition; (5) Patients voluntarily participated and signed the informed consent form. The exclusion criteria were as follows: (1) Male patients; (2) Patients with comorbid serious mental or neurological diseases who are unable to complete the survey.

### Survey tools

This survey consists of five parts: general demographic characteristics, the Social Impact Scale (SIS), the Perceived Social Support Scale (PSSS), the Breast Cancer Resilience Scale (BCRS), and the MOS Item Short Form Health Survey (SF-36).

The general demographic characteristics included age, educational level, marital status, profession, monthly income per capita, insurance payment mode, number of offspring, main caregiver, and duration of the disease.

The SIS scale was developed by Fife et al.^19^, and sinicized by Pan et al.^20^. It was used to assess the discrimination degree perceived by individuals, from 4 dimensions and 24 items: economic discrimination (3 items), social exclusion (9 items), social isolation (7 items), and intrinsic shame (5 items). Each item employed a Likert 4-point scale, with responses ranging from “Strongly Disagree” (1 score) to “Strongly Agree” (4 scores), yielding total scores from 24 to 96. A higher score indicates a higher level of perceived discrimination. Its Cronbach’s α = 0.86.

The PSSS scale was developed by Zimet et al.^21^, which was used to assess the social support perceived by individuals. The scale consists of 3 dimensions and 12 items: family support (4 items), friend support (4 items), and other support (4 items). Each item employed a Likert 7-point scale, with responses ranging from “not at all compliant” (1 score) to “fully compliant” (7 scores), yielding total scores from 12 to 84. A higher score indicates a higher level of social support. Its Cronbach’s α = 0.88.

The BCRS scale was developed by Sunaga et al.^22^ and was used to specifically assess the adverse feeling and psychological resilience of breast cancer patients. It consists of 2 dimensions and 16 items: personal protection (11 items) and social protection (5 items), Each item employed a Likert 4-point scale, with responses ranging from “completely disagree” (1 score) to “absolutely agree” (4 scores), yielding total scores from 16 to 64. A higher score indicates greater psychological resilience in breast cancer patients. Its Cronbach’s α = 0.93, and its retest correlation coefficient = 0.89.

The SF-36 scale was developed by the Medical Outcomes Study (MOS)^23^. It consists of 8 dimensions: physical functioning (PF), role-physical health (RP), bodily pain (BP), general health status (GH), vitality (VT), social functioning (SF), role-emotional problem (RE), and mental health (MH). This scale consists of 36 items. The scoring for each item is relatively complex; however, each dimension is standardized to a score out of 100, and the total score for the SF-36 is 800. These dimensions cover both physical and mental health aspects to reflect the overall QoL of patients^24^. The Cronbach’s α of the SF-36 scale is 0.80.

### Survey procedure and quality control

This survey utilized a *Wenjuanxing* QR code for the collection of questionnaire data. Prior to conducting the survey, all investigators underwent standardized training to ensure the completeness and accuracy of the questionnaire responses. Following one-on-one communication and detailed explanations provided to the patients, the investigators distributed the QR codes, enabling patients to complete the questionnaires independently. For patients who were unable to complete the questionnaire on their own, assistance from family members was allowed to facilitate completion.

To ensure quality control, the distribution and collection of questionnaires were managed by designated staff members. All questionnaires were distributed on-site and promptly retrieved for verification. In cases where patients had questions or required clarification, investigators offered standardized instructions or necessary clarification to minimize the percentage of invalid questionnaires. During the data entry phase, Epi Data 3.1 was utilized for double data entry conducted by two trained personnel to ensure the precision and reliability of the entered data.

### Statistical analysis

The statistical analysis was performed on the IBM SPSS 27.0 software (SPSS, USA) and IBM SPSS Modeler 18.0 software (SPSS, USA). The significance level is set as α = 0.05 (two-sided). Count data were expressed as frequencies and percentages [n(%)], while measurable data were described as mean ± standard deviation ($\bar{x}$ ± SD). The differences in SF-36 scores across demographic characteristics groupings were analyzed using either an independent samples t-test or analysis of variance (ANOVA), as appropriate. The bivariate correlations between SIS, PSSS, BCRS, and SF-36 scores were analyzed via Spearman correlation heat-plot, with |r|≥0.3 as the criterion for clinically acceptable evident correlation^25^. When *P* < 0.05, the |r| of ≤ 0.1, > 0.1 to 0.3, > 0.3 to 0.5, > 0.5 to 0.7, > 0.7 to 0.9, and > 0.9 were considered to indicate no correlation, poor correlation, mild correlation, moderate correlation, strong correlation, and extremely strong correlation, respectively. The multiple linear regression analyzed the independent associations between SF-36 scores and the other survey items, with β ± 1.96SE < 0 and β ± 1.96SE > 0 as the criterion for independent negative and positive correlation when *P* < 0.05, respectively. The mediation effect analysis was conducted in IBM SPSS Modeler 18.0 software.

## Results

### The scores of 4 questionnaires and subgroup analysis of the SF-36 scale

**Fig. 1:**
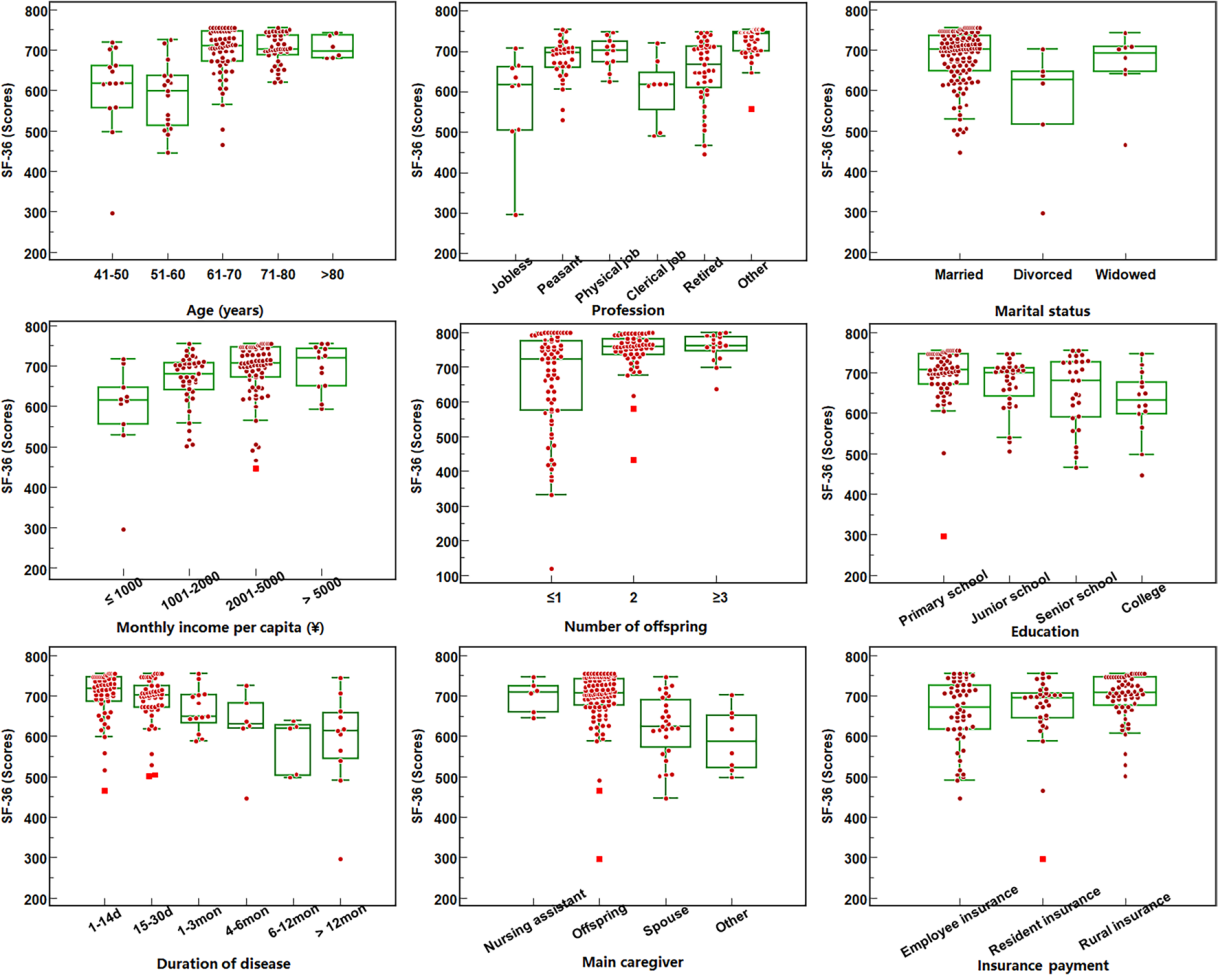
SF-36 scores across different demographic characteristic groupings. note: Significant differences were found in the SF-36 scores among different groupings.

A total of 150 patients participated in this study. After excluding 5 incomplete responses and 2 erroneous responses, 143 valid responses were analyzed (recovery rate: 95.3%). Their demographic characteristics are presented in Supplementary Table 1. The scores of four scales were as follows: SIS = 46.2 ± 16.1, PSSS = 73.2 ± 9.9, BCRS = 54.0 ± 7.7, and SF-36 = 745.5 ± 58.5 (More details are shown in Table 1). After grouping by various demographic characteristics, the ANOVA test revealed significant differences in SF-36 scores among groups defined by age, education level, marital status, profession, monthly income per capita, insurance payment mode, number of offspring, main caregiver, and disease duration groups (F = 3.960–15.734.960.734, all *P* < 0.05) (Shown in Fig. 1).

**Table 1:** Total and dimensional scores of the SIS, PSSS, BCRS and SF-36 scales among elderly patients with breast Cancer.

Scale	Median (Min, Max)	Mean ± SD
SIS	46 (24, 85)	46.2 ± 16.1
Economic discrimination	6 (3, 12)	6.2 ± 2.7
Social exclusion	17 (9, 34)	15.9 ± 6.5
Social segregation	13 (7, 24)	12.5 ± 5.2
Intrinsic shame	12 (5, 19)	11.6 ± 3.5
**PSSS**	**76 (39**,** 84)**	**73.2 ± 9.9**
Family support	28 (16, 28)	26.8 ± 2.8
Friend support	24 (10, 28)	23.8 ± 4.4
Other support	24 (8, 28)	22.5 ± 4.8
**BCRS**	**53 (37**,** 64)**	**54.0 ± 7.7**
Personal protection	40 (22, 44)	38.5 ± 5.4
Social protection	15 (5, 20)	15.6 ± 4.00
**SF-36**	**743 (300**,** 750)**	**745.5 ± 58.8**
Physical functioning	95 (35, 95)	88.0 ± 11.8
Role physical	95 (35, 95)	83.9 ± 30.1
Bodily pain	100 (41, 100)	92.9 ± 14.3
General health	85 (20, 85)	75.5 ± 17.3
Vitality	90 (30, 90)	80.6 ± 15.8
Social Function	95 (65, 95)	87.1 ± 16.5
Role emotional	95 (42, 95)	86.2 ± 27.4
Mental health	88 (32, 95)	80.9 ± 16.2

Note: SIS, Social Impact Scale; PSSS, Perceived Social Support Scale; BCRS, Breast Cancer Resilience Scale; SF-36, Short Form Health Survey.

### Bivariate correlation between SF-36 and SIS/PSSS/BCRS scores

**Fig. 2:**
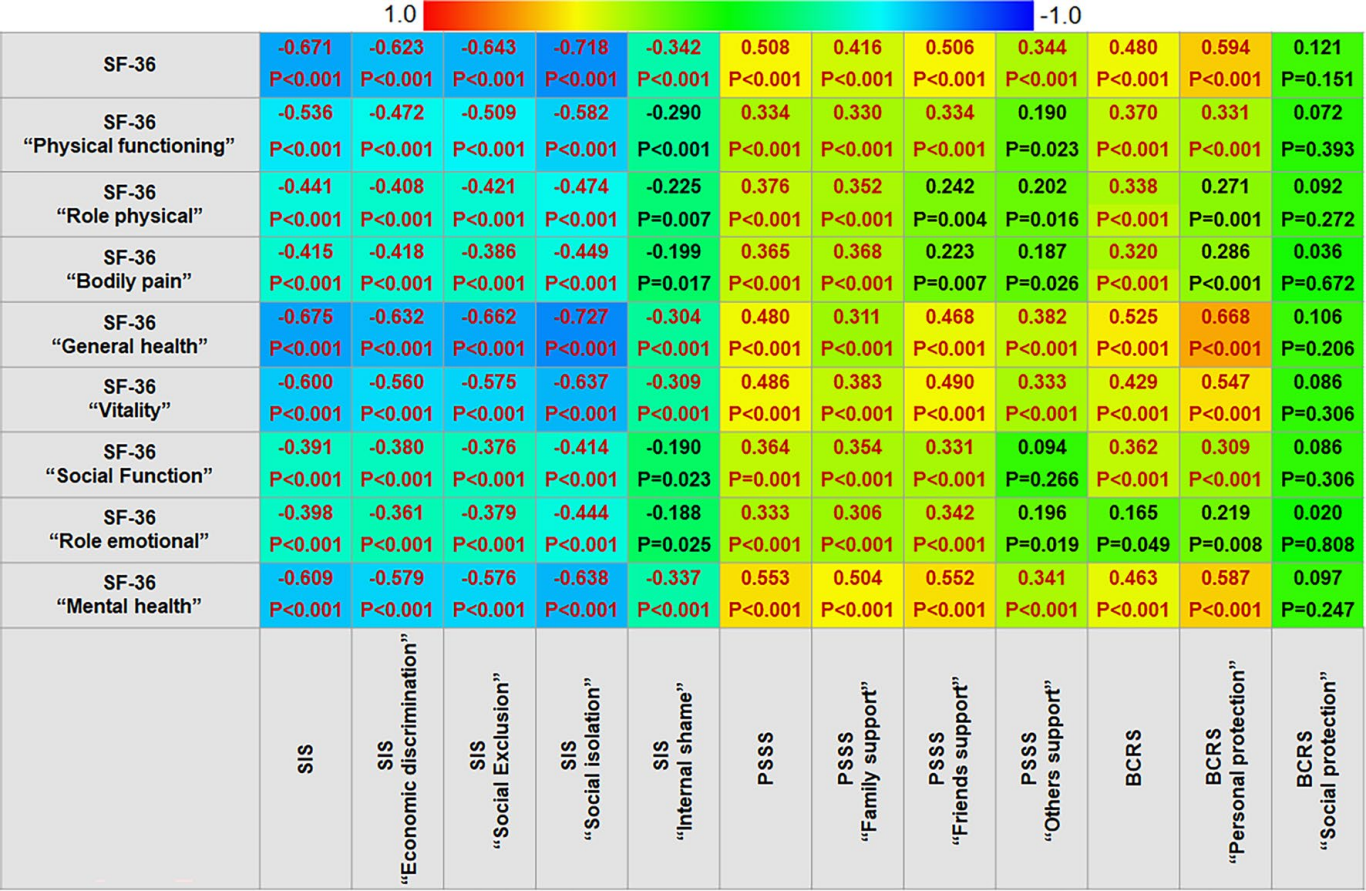
Bivariate correlation between SF-36 and SIS/PSSS/BCRS total and dimensional scores. note: SF-36, Short Form Health Survey; SIS, Social Impact Scale; PSSS, Perceived Social Support Scale; BCRS, Breast Cancer Resilience Scale. SIS/PSSS/BCRS. Overall, the SF-36 scores were negatively correlated with the SIS scores, but positively correlated with the PSSS, and BCRS scores, except for the BCRS “social protection” scores. Between the dimensional scores, the SF-36 “physical functioning”, “general health”, “vitality”, and “mental health” scores presented moderate to strong correlations with nearly all SIS dimensional scores (*r*=−0.509 to 0.727, all *P* < 0.001), with the highest correlation observed between the SF-36 “general health” and the SIS “social isolation” scores (*r*=−0.727, *P* < 0.001); the SF-36 “mental health” scores presented moderate correlations with the PSSS “family support” and “friend support” scores as well as the BCRS “personal protection” scores (*r* = 0.504, 0.552, and 0.587, all *P* < 0.001), and the SF-36 “general health”, and “vitality” scores also presented moderate correlations with the BCRS “personal protection” scores (*r* = 0.668 and 0.547, both *P* < 0.001). An evident correlation, using |r|>0.3 as the criteria^25^, is shown in red font.

The Pearson correlation heat-plot illustrated the bivariate correlations between SF-36 and SIS/PSSS/BCRS scores. Overall, the SF-36 scale scores demonstrated negative correlations with SIS scale scores, whereas positive correlations with PSSS and BCRS scale scores (all *P* < 0.05), except for the BCRS “social protection” dimensional score (Shown in Fig. 2). When a criterion of |r|>0.3 was applied to indicate evident correlations, it was found that mild to strong correlations retained between SF-36 total and dimensional scores with SIS, PSSS, and BCRS total scores (except between SF-36 “role emotional” and BCRS; |r|=0.320 ~ 0.668, all *P* < 0.001), as well as with most of their dimensional scores (|r|=0.304 ~ 0.727, all *P* < 0.001). The statistical power of all evident correlation coefficients ranged from 0.747 to 1.000; only when |r| exceeded 0.370, the power reached 0.901. Of them, the SF-36 “general health” scores showed the highest negative correlation with the SIS “social isolation” scores (*r*=−0.727, *P* < 0.001), but the highest positive correlation with the BCRS “personal protection” scores (*r* = 0.668, *P* < 0.001).

### Independent associations of SF-36 with SIS, PSSS, and BCRS

The stepwise multiple linear regression analysis demonstrated an independent and negative association of SIS “social isolation” dimensional score with SF-36 overall score and its dimensional scores (β=−0.82~−13.50, all *P* < 0.05) (Shown in Table 2). Additionally, the BCRS “personal protection” dimensional score was independently and positively correlated with SF-36 overall score and its “general health”, “vitality”, and “mental health” dimensional scores (β = 0.64 ~ 5.74, all *P* < 0.05). The PSSS “family support” dimensional score was positively correlated with the SF-36 “social function” and “mental health” dimensional scores (β = 0.93 and 1.27, both *P* < 0.05).

**Table 2:** Independent influences of SIS, PSSS and BCRS dimensions on SF-36 dimensions.

Score	SIS3_Social isolation		BCRS1_Personal protection		PSSS1_Family support	
	β ± SE	t, *P*	β ± SE	t, *P*	β ± SE	t, *P*
SF-36 scale	−13.50 ± 1.56	−8.674, < 0.001	5.74 ± 2.85	2.013, 0.046	ND	ND
SF-36 “Physical functioning”	−2.12 ± 0.51	−4.164, < 0.001	ND	ND	ND	ND
SF-36 “Role physical”	−3.98 ± 1.43	−2.794, 0.007	ND	ND	ND	ND
SF-36 “Bodily pain”	−1.89 ± 0.68	−2.760, 0.007	ND	ND	ND	ND
SF-36 “General health”	−2.13 ± 0.59	−3.615, < 0.001	1.22 ± 0.24	5.001, < 0.001	ND	ND
SF-36 “Vitality”	−1.82 ± 0.64	−2.863, 0.005	0.64 ± 0.26	2.430, 0.016	ND	ND
SF-36 “Social function”	−0.82 ± 0.31	−2.684, 0.008	ND	ND	0.93 ± 0.39	2.382, 0.019
SF-36 “Role emotional”	−4.52 ± 1.30	−3.469, < 0.001	ND	ND	ND	ND
SF-36 “Mental health”	−1.42 ± 0.61	−2.330, 0.021	0.78 ± 0.25	3.080, 0.002	1.27 ± 0.42	3.045, 0.003

note: ND, not included in the regression equation due to *P* > 0.10.

### Independent influencing factors of QoL among elderly breast cancer patients

Taking the SF-36 total score as the dependent variable, the SIS, PSSS, and BCRS dimensional scores and demographic characteristics as potential influencing factors, the stepwise multivariate regression analysis revealed close associations of SIS “social isolation” (r_partial_=−0.357, *P* < 0.001), patient age (r_partial_=0.258, *P* = 0.002), disease duration (r_partial_=−0.189, *P* = 0.025), and BCRS “personal protection” (r_partial_=0.179, *P* = 0.045) with the QoL of elderly breast cancer patients (Table 3). Among them, BCRS “personal protection” score and patient age were protective factors (β = 4.03 and 26.83, both *P* < 0.05), whereas SIS “social isolation” score and disease duration were risk factors (β=−9.01 and − 12.22, both *P* < 0.05).

**Table 3:** Stepwise multiple linear regression of factors influencing the quality of life in elderly breast cancer patients.

Independent variables	β	SE	t	*P*	*r*_partial_	VIF
BCRS “Personal protection”	4.08	2.01	2.205	0.045	0.179	1.737
SIS “Social isolation”	−9.01	2.01	−4.488	< 0.001	−0.357	2.247
Disease duration	−12.22	5.41	−2.261	0.025	−0.189	1.294
Patient age	26.83	8.54	3.141	0.002	0.258	1.472

note: The dependent variable is the SF-36 total scores, and the covariates are demographic characteristic information as well as the SIS, PASS and BCRS dimensional scores.

### PSSS enhanced BCRS to alleviate the effect of SIS on SF-36

**Fig. 3:**
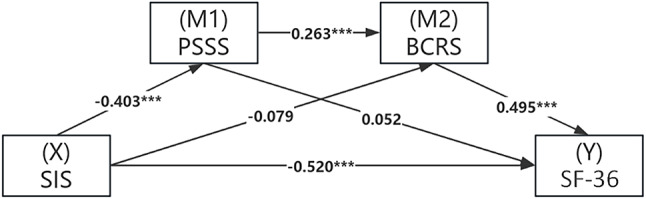
Mediating effects of PSSS and BCRS on the regulation of SF-36 by SIS. Note: SF-36, Short Form Health Survey; SIS, Social Impact Scale; PSSS, Perceived Social Support Scale; BCRS, Breast Cancer Resilience Scale. The regulation roles are significant only in two pathways: (1) SIS directly and negatively affected SF-36; and (2) SIS indirectly and negatively affected SF-36 through the mediating effect of the interaction between PSSS and BCRS.

A structural equation modeling was constructed with SIS as the independent variable, PSSS and BCRS as the mediating variables, and SF-36 as the dependent variable. A bootstrapping was conducted with 1,000 resamples to assess the robustness of the mediation effects. The SIS significantly affected SF-36 through two pathways (Fig. 3; Table 4): (1) a direct pathway, wherein SIS negatively predicted SF-36, contributing 82.28% out of total effect (β=−0.520, Bootstrap 95%CI=−0.668 to −0.371, *P* < 0.001); and (2) an indirect pathway, that is a mediating pathway of interaction between PSSS and BCRS, wherein SIS negatively predicted PSSS (β=−0.403, *P* < 0.001), followed by PSSS positively predicted BCRS (β = 0.263, *P* < 0.001), and finally BCRS positively predicted SF-36 (β = 0.495, *P* < 0.001), contributing 8.39% out of total effect (β=−0.053, Bootstrap 95%CI=−0.098 to −0.019, *P* < 0.001). However, neither PSSS nor BCRS alone significantly mitigated the effect of SIS on SF-36, although contributing 3.32% and 6.17% out of total effect, respectively (both *P* > 0.05).

**Table 4:** The mediating effect of PSSS and/or BCRS on the regulation of SF-36 by SIS (Bootstrap = 1000 times).

Effect types	Effect	Boot SE	Bootstrap 95%(CI)		Relative mediation effect
			Boot LLCI	Boot ULCI	
Total effects	−0.632	0.059	−0.749	−0.516	-
Direct effects (X→Y)	−0.520	0.075	−0.668	−0.371	82.28%
Total Indirect effects	−0.113	0.060	−0.236	−0.001	17.88%
Indirect effects1 (X→M1→Y)	−0.021	0.062	−0.142	0.099	3.32%
Indirect effects2 (X→M2→Y)	−0.039	0.029	−0.102	0.010	6.17%
Indirect effects3 (X→M1→M2→Y)	−0.053	0.020	−0.098	−0.019	8.39%

note: X, SIS; M1, PSSS; M2, BCRS; Y, SF; SE, standard error; CI, confidence interval; LLCI, lower limit of confidence interval; ULCI, upper limit of confidence interval.

## Discussion

This study highlights the significant impact of perceived social support and psychological resilience in elderly breast cancer patients between social support and their QoL. Our findings suggest that patient’s perceived social support mediates the effect of external social support on QoL by enhancing psychological resilience. A good perception of social support and strong psychological resilience positively contribute to maintaining a higher QoL. Specifically, personal protection ability and social isolation status are closely related to their QoL. Overall, their perceived social support and psychological resilience significantly mediate the negative effects of social factors on QoL. Additionally, patient age and disease duration also strongly influence their QoL.

Our study investigates the contemporary status of social support, perceived social support, psychological resilience, and QoL among elderly breast cancer patients. Unlike related studies focusing on elderly individuals living alone or patient populations with other specific diseases, our findings reveal distinct differences^26,27^. In this investigation, the mean SIS score of 46.17 indicates a moderate level of influence from the social environment on patients. However, the mean PSSS score of 73.22 reflects a high level of perceived social support among elderly breast cancer patients. Of the three dimensions of PSSS, support from sources other than family and friends scores the lowest, suggesting that elderly patients may lack adequate perceived social support beyond family and friend sources. This deficiency can be attributed to the contraction of their social circle or less frequent social interactions. In contrast, patients with elderly breast cancer tend to receive increased humanistic care and support from family and friends due to the empathy elicited by their condition. The mean BCRS score of 54.04 is also at a high level, indicating that elderly patients demonstrate strong adaptability and resilience in coping with stresses and challenges^27^. The mean SF score of 745.5 is at a medium-high level, which suggests that elderly patients generally maintain a relatively good overall QoL but still have room for improvement.

Subsequently, after finding that all nine sociodemographic characteristics investigated are significantly associated with QoL, we incorporate them into the multiple correlation analysis to identify key areas for targeted interventions. Our findings reveal that patient age and disease duration are independent influences on their QoL. When formulating treatment plans for patients with breast cancer, age represents one of the critical factors to consider. Older patients often experience declining physical function and immune function, reducing their ability to tolerate intensive treatments like chemotherapy or radiotherapy. As a result, clinicians may choose more conservative approaches, which may result in suboptimal disease control and enhanced susceptibility to adverse effects such as myelosuppression and neurotoxicity. These issues can worsen physical function and negatively affect QoL^28,29^. Therefore, patient age influences both treatment choices and QoL through its impact on treatment tolerance. Regarding the disease duration, it represents a critical factor that significantly impacts the physical function and QoL of patients. Studies have demonstrated that as the duration of the disease extends, there is a progressive decline in patients’ physical function of patients, along with a corresponding decrease in their QoL. Therefore, early detection and treatment are crucial for improving the QoL of elderly breast cancer patients^30^. These two sociodemographic factors will become key focal points in the care of breast cancer patients. For elderly patients with a prolonged disease course, it is recommended to encourage greater participation in social activities. This approach not only helps prevent social isolation and alleviate feelings of loneliness but also facilitates broader social recognition and emotional support.

In addition to the above two demographic characteristics of patients, our study also reveals that patients’ personal protection ability and social isolation status are independent influences on their QoL. The strength of the social support system positively impacts patients’ emotions and life adaptation, leading to more stable emotions and a higher QoL, which is crucial for improving the QoL in elderly breast cancer patients^31^. Social isolation represents a social state in which the connections with family, friends, and society are diminished for elderly breast cancer patients. Studies have indicated that psychological or physical disconnection between patients and others may result in subjective experiences of lacking belongingness or intimacy^32–34^, and can even elevate the risk of depression and anxiety^35^. Psychological resilience refers to the capacity of patients to psychologically recover from stress and adversity, and personal protection refers to the psychological protection presented by patients in response to the prolonged disease process, changes in their personal signs, or other f influencing actors^36^. Studies have demonstrated that breast cancer patients can reduce their excessive self-protection through positive psychological suggestions, seeking social support, or participating in rehabilitation activities, thus alleviating their psychological stress^37–39^. So, the improvement of psychological resilience is an effective strategy to improve their QoL. In summary, the QoL for elderly breast cancer patients can be effectively improved through strengthened support from primary caregivers, early detection and timely intervention, a robust social support network, and the cultivation of psychological resilience.

The multivariate analysis conducted in our study confirms significant associations of the SF-36 total and its multidimensional scores with the scores of the social isolation dimension in the SIS scale and personal protection dimension in the BCRS scale, alongside the family support dimension in the PSSS scale. Specifically, the social isolation scores are strongly correlated to the SF-36 total scores and each of its dimensional scores; the personal protection scores are strongly correlated to the scores of the “general health”, “vitality”, and “mental health” dimensions. These findings suggest that the impact of social support on the QoL of breast cancer patients is comprehensive and extensive; furthermore, maintaining general health, mental health, and vitality appears to be crucial for enhancing their psychological resilience. In addition, our study also identifies a significant association of the family support scores with SF-36 total scores and its dimensional score of “social function” and “mental health”. Consistent with prior research, adequate family support has been demonstrated to effectively mitigate patients’ negative emotions and strengthen their capacity to manage their disease^40^. Family support serves not only as a critical safeguard for patients’ psychological well-being but also as a fundamental pillar for enhancing overall QoL.

Ultimately, the mediation effect analysis confirms that the interaction between patients’ perceived social support and their psychological resilience exhibited a significant mediation effect. Our findings reveal a direct negative impact of SIS scores on both SF-36 and PSSS scores, underscoring the detrimental effects of adverse social factors (e.g., social exclusion, economic discrimination, internalized shame, and social isolation) on the QoL and perceived social support.

Despite an insignificant effect on QoL found in our mediating model, perceived social support exhibits a significant positive effect on psychological resilience. Previous studies have consistently shown that psychological resilience positively contributes to the QoL among elderly breast cancer patients^41^; Meanwhile, low levels of perceived social support may intensify an inadequate psychological resilience, ultimately leading to a further decline in QoL^42,43^. Thus, our mediating model highlights the critical role of interaction between perceived social support and psychological resilience in improving the QoL for elderly breast cancer patients, offering a feasible theoretical foundation for further clinical intervention and treatment. Therefore, Consequently, reinforcing social support and enhancing social support perception can bolster their psychological resilience, ultimately leading to an improvement in their QoL.

## Conclusions

Our findings crudely suggest that the interaction between perceived social support and psychological resilience in elderly breast cancer patients serves as a mediating factor in the negative adjustment of social support to patients’ QoL. These findings offer valuable insights for optimizing clinical practice, nursing care, and psycho-oncological support for breast cancer patients. When follow-up duration, nurses should assess their understanding of the illness and treatment, along with any self-protection measures they have taken, then provide personalized self-care guidance and psychological education. Especially, help patients recognize and change irrational beliefs about “personal protection” to encourage more balanced coping strategies, assess social support and signs of isolation—such as living alone or avoiding social contact—and involve family and friends in care and social engagement. Nurses should also help patients address emotional barriers like shame or body image concerns to support social reintegration. These insights emphasize the importance of a patient-centered, interdisciplinary care approach.

The limitation of this study: a single-center and small-sample survey, alongside ethnic and regional differences, limits the extrapolation of conclusions to some extent. The test power of certain indicators in both Spearman correlation analysis and linear regression analysis fell short of the 0.9 threshold, suggesting that the conclusions regarding their association with SF-36 require further validation. Moreover, self-report bias and potential selection bias cannot be entirely avoided, which may introduce discrepancies when comparing the findings with those of other studies. Additionally, the cross-sectional design precludes the establishment of causal relationships. Future studies should focus on multi-center, large-sample, and longitudinal designs to recruit a greater number of elderly breast cancer patients, so as to confirm the generalizability and reliability of our findings. A more comprehensive exploration of factors influencing the QoL in this population can facilitate the development of targeted interventions, ultimately improving patient outcomes.

## Supplementary Information

Below is the link to the electronic supplementary material.

Supplementary Material 1

## Data Availability

The datasets used during this study are available from the corresponding author on reasonable request.
