# Supplementary material for: Perceived support and psychological resilience as collaborative mediators between external support and quality of life in elderly breast cancer patients
Source: Sci Rep. 2025 Nov 11;15:39541. doi: 10.1038/s41598-025-23246-x (PMC12606279; doi:10.1038/s41598-025-23246-x)
Supplement: Supplementary file 1 — Supplementary Material 1 [file 41598_2025_23246_MOESM1_ESM.docx]

**Supplementary Table 1. The demographic characteristics of 143 participant**

| **Item** | **Category** | **Frequency (n)** | **Percentage (%)** |
| --- | --- | --- | --- |
| Age | 40-50 years old | 14 | 9.8 |
|  | 51-60 years old | 17 | 11.9 |
|  | 61-70 years old | 63 | 44.1 |
|  | 71-80 years old | 43 | 30.1 |
|  | Above 80 years old | 6 | 4.2 |
| Education | Primary school | 70 | 49 |
|  | Junior school | 31 | 21.7 |
|  | Senior school | 28 | 19.6 |
|  | College | 14 | 9.8 |
| Marital status | Married | 129 | 90.2 |
|  | Divorced | 6 | 4.2 |
|  | Widowed | 8 | 5.6 |
| Profession | Jobless | 9 | 6.3 |
|  | Peasant | 33 | 23.1 |
|  | physical job | 13 | 9.1 |
|  | Clerical job | 6 | 4.2 |
|  | Retired | 39 | 27.3 |
|  | Other | 43 | 30.1 |
| Monthly income per capita | ≤1000 ¥ | 10 | 7 |
|  | 1001-2000 ¥ | 41 | 28.7 |
|  | 2001-5000 ¥ | 79 | 55.2 |
|  | ≥5001 ¥ | 13 | 9.1 |
| Number of offspring | ≤1 | 67 | 46.8 |
|  | 2 | 58 | 40.6 |
|  | ≥3 | 18 | 12.6 |
| Main caregiver | Nursing assistant | 6 | 4.2 |
|  | Offspring | 101 | 70.6 |
|  | Spouse | 28 | 18.9 |
|  | Other | 8 | 5.6 |
| Duration of disease | 1-14 d | 54 | 37.8 |
|  | 15-30 d | 54 | 37.8 |
|  | 1-3mon | 13 | 9.1 |
|  | 4-6mon | 6 | 4.2 |
|  | 6-12mon | 5 | 3.5 |
|  | > 12mon | 11 | 7.7 |
| Insurance payment | Employee insurance | 52 | 36.4 |
|  | Resident insurance | 31 | 21.7 |
|  | Rural insurance | 60 | 41.9 |
